# A CIE change in our understanding of endocytic mechanisms

**DOI:** 10.3389/fcell.2023.1334798

**Published:** 2023-12-13

**Authors:** Daniel J. Rioux, Derek C. Prosser

**Affiliations:** ^1^ Life Sciences, Virginia Commonwealth University, Richmond, VA, United States; ^2^ Department of Biology, Virginia Commonwealth University, Richmond, VA, United States

**Keywords:** endocytosis, clathrin-independent, clathrin, model organism, actin, small GTPase

## Abstract

The past six decades have seen major advances in our understanding of endocytosis, ranging from descriptive studies based on electron microscopy to biochemical and genetic characterization of factors required for vesicle formation. Most studies focus on clathrin as the major coat protein; indeed, clathrin-mediated endocytosis (CME) is the primary pathway for internalization. Clathrin-independent (CIE) pathways also exist, although mechanistic understanding of these pathways remains comparatively elusive. Here, we discuss how early studies of CME shaped our understanding of endocytosis and describe recent advances in CIE, including pathways in model organisms that are poised to provide key insights into endocytic regulation.

## 1 Introduction

As we approach 2024, we mark 60 years of structural, biochemical, and genetic studies of endocytosis. While early work reported observations of phagocytosis and macropinocytosis, Roth and Porter capitalized on advances in electron microscopy to visualize mosquito oocytes forming pits at the plasma membrane (PM), resulting in intracellular vesicles (Roth and Porter, 1964). Their 1964 work suggested events leading to internalization: 1) assembly of a “bristle coat” at the PM, 2) invagination of bristle-coated structures into pits, 3) conversion of pits into vesicles, and 4) loss of the coat. Roth and Porter proposed mechanical functions for the coat that were later verified, including in a key 1969 study by Kanaseki and Kadota describing the coated structure as “*The Vesicle in the Basket*” (Kanaseki and Kadota, 1969). Here, electron micrographs of bristle-coated vesicles isolated from guinea pig brain demonstrated a polygonal coat surrounding the vesicle. Coat formation progressed from shallow, bristle-lined pits on the cytoplasmic face to the completed vesicle. Subsequently, Barbara Pearse isolated coated vesicles from pig brain in 1975, and described a polygonal structure consistent with findings from Kanaseki and Kadota (Pearse, 1975). Pearse purified a 180 kDa protein as the sole component of coats, and named it clathrin. These and other foundational papers accurately described the process of clathrin-mediated endocytosis (CME), identified the key coat protein, and formed the basis of our modern understanding of endocytosis.

CME is the main pathway for PM internalization (Kaksonen and Roux, 2018). It is currently the best-studied endocytic route, and involves sequential action of protein modules to recruit cargo, deform the PM, and generate a clathrin-coated vesicle (CCV) (Kaksonen et al., 2005; Newpher et al., 2005). During initial stages of clathrin-coated pit (CCP) formation, which may occur stochastically or from initiating cues, early-arriving proteins such as FCHo1/2 and the clathrin-binding adaptor complex AP-2 associate with endocytic sites by binding phosphoinositides, particularly phosphatidylinositol 4,5-bisphosphate [PI(4,5)P_2_] (Howard et al., 2002; Henne et al., 2010; Cocucci et al., 2012). Adaptors serve a dual function in cargo-binding and in providing an anchor point for clathrin assembly. The clathrin lattice consists of heavy and light chains that interact to form a triskelion (Unanue et al., 1981). As the CCP matures, scaffolding proteins recruit later-acting factors that facilitate invagination of endocytic pits, often through activation of Arp2/3 (Kübler and Riezman, 1993; Kaksonen et al., 2003; 2005; Sun et al., 2006; Goode et al., 2015). Finally, Bin/amphiphysin/Rvs (BAR)-domain proteins such as amphiphysin, syndapin and SNX9, and F-BAR proteins FBP17 and CIP4 interact with the GTPase dynamin at the CCP neck to facilitate scission (Bliek et al., 1993; Damke et al., 1994; Takei et al., 1995; Qualmann and Kelly, 2000; Kamioka et al., 2004; Soulet et al., 2005; Shimada et al., 2007; Yu and Yoshimura, 2022). Separated from the PM, the CCV quickly loses its coat following recruitment of auxilin/DnaJ, Hsp70, and synpatojanins (Ungewickell et al., 1995). Uncoated vesicles are then free to fuse to their target compartments. CME is conserved in eukaryotes, and many endocytic proteins perform similar functions in all species examined to date (Taylor et al., 2011).

**FIGURE 1:**
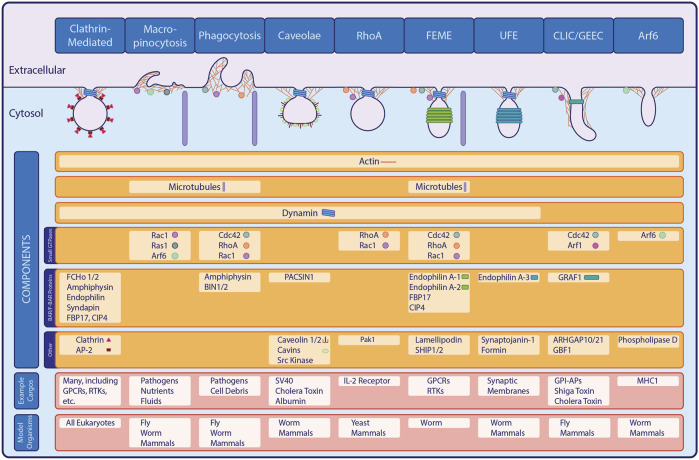
Major Forms of Endocytosis. In addition to the well-studied clathrin-mediated endocytic pathway, a variety of clathrin-independent pathways exist in eukaryotes. Shown here, many of the known CIE pathways share overlapping machinery or classes of proteins with CME or with each other, while also possessing unique components and internalizing specific cargo proteins.

Aside from CME, other pathways perform endocytosis without the clathrin coat. Collectively termed clathrin-independent endocytosis (CIE), these pathways offer additional routes for cargo entry (Figure 1). Indeed, early studies predating discovery of the bristle coat described phagocytosis and macropinocytosis, which are inherently clathrin-independent (Tauber, 2003; King and Kay, 2019). Intriguingly, molecular mechanisms of CIE remain poorly understood compared to CME. Recent studies advancing our understanding of CIE are filling gaps in our knowledge, with model organisms permitting identification of CIE genes that are likely conserved. In this review, we examine the history of CIE research, explore recent discoveries, and look toward new questions in the field.

### 1.1 Clathrin-independent endocytosis

Although CCV purification permitted identification of CME machinery proteins, evidence of additional pathways predated the discovery of coated pits (Keen et al., 1979; Zaremba and Keen, 1983). For example, phagocytosis and micropinocytosis were first observed over 100 years ago, while caveolae were described nearly a decade before the discovery of coated vesicles but were not confirmed as endocytic structures until later (Tauber, 2003; King and Kay, 2019). Studies in the 1980s described formation of uncoated endocytic carriers, suggesting that CIE exists distinct from (and/or parallel to) CME (Moya et al., 1985). A clathrin-independent pathway was finally demonstrated in the 1990s with experiments that showed uptake of ricin persisted upon inhibition of transferrin internalization via CCVs (Sandvig and Deurs, 1990; Hansen et al., 1991). Additionally, genetic and biochemical studies showed that cells expressing dominant-negative mutants of CME proteins such as dynamin, epsinR, eps15 and AP180 still internalized fluid-phase and PM components (Damke et al., 1994; Benmerah et al., 1998; Chen et al., 1998; Ford et al., 2001). Identification of additional, mechanistically-distinct CIE pathways further demonstrated that endocytosis is far more complex than originally thought (Figure 1). In addition to phagocytic, macropinocytic and caveolar routes, these include clathrin-independent carriers and GPI-enriched endocytic compartments (CLIC/GEEC), small GTPase-mediated pathways, fast endophilin-mediated endocytosis (FEME) and ultrafast endocytosis (UFE) (Lamaze et al., 2001; Kirkham et al., 2005; Lundmark et al., 2008; Watanabe et al., 2013; Boucrot et al., 2015; Hak et al., 2018; Sathe et al., 2018; Casamento and Boucrot, 2020; Imoto et al., 2022).

#### 1.1.1 Phagocytosis and macropinocytosis

Phagocytosis and macropinocytosis were amongst the earliest forms of endocytosis observed; both internalize large volumes in a single event. Phagocytosis occurs when cells extend the PM around particles to engulf them. This mechanism is highly conserved from protists such as *Dictyostelium discoideum* to immune cells (e.g., neutrophils and macrophages), and internalizes nutrients, clears debris and dead cells, and combats pathogens (Tauber, 2003; Vorselen et al., 2020). Initiation of a phagocytic cup occurs when surface receptors such as integrins recognize molecules on the foreign particle, triggering recruitment and activation of RhoA, Rac1 and Cdc42 along with actin modulators such as WASP and Arp2/3 (Caron and Hall, 1998; Jaumouillé et al., 2019). This machinery drives formation of pseudopodia through assembly of actin networks that provide force to drive PM protrusion. Similarly, macropinocytosis is actin-dependent, and relies on Rac1, Ras and Arf6 to induce PM ruffling that internalizes extracellular fluids, nutrients, growth factors and pathogens (Bar-Sagi and Feramisco, 1986; Fujii et al., 2013). Aside from bulk internalization, macropinocytosis allows cells to explore their environment and receive signals from other cells (Veltman et al., 2016). Closure and scission of phagosomes and macropinosomes remains poorly understood; however, myosins likely play a role through interactions with actin (Brzeska et al., 2016).

#### 1.1.2 Caveolar endocytosis

Caveolae are detergent-resistant, flask-shaped invaginations found in PM regions with high cholesterol and sphingolipid levels (Parton et al., 2006). The structure of caveolae is determined by the transmembrane proteins caveolin-1 (CAV1) and caveolin-2 (CAV2), which form a heterodimer that interacts with cytosolic cavins (Kiss and Botos, 2009). Caveolae are generally immobile, but binding of ligands such as simian virus 40, cholera toxin or albumin induces internalization (Pelkmans et al., 2002). Upon ligand binding, CAV1 recruits Src-family tyrosine kinases to specialized scaffolding domains. Src then phosphorylates CAV1/2, leading to endocytosis through currently-unclear mechanisms that depend on dynamin (Oh et al., 1998; Shajahan et al., 2004; Sverdlov et al., 2007).

#### 1.1.3 Small GTPase-Dependent CIE

To date, several CIE pathways involving Rho- and Arf-family GTPases have been identified but remain understudied. RhoA-dependent CIE internalizes the interleukin-2 receptor (IL2R); this pathway is dynamin-dependent, and requires Rac1 and its effector Pak1 (Lamaze et al., 2001). RhoA-dependent CIE is also responsible for compensatory endocytosis in bladder umbrella cells, which rapidly expand during bladder filling and contract during voiding. RhoA-dependent CIE allows for rapid PM internalization after voiding, likely in response to loss of membrane tension (Khandelwal et al., 2010). CIE in umbrella cells requires integrins but not Rac1, suggesting mechanistic distinctions from IL2R internalization.

Arf6 defines a dynamin-independent CIE route, that internalizes MHC1 as its major cargo, wherein the GTPase contributes to actin polymerization, activation of phosphatidylinositol-4-phosphate 5-kinase (PIP5K) and phospholipase D (PLD) (Naslavsky et al., 2004). PIP5K then generates PI(4,5)P_2_, which is required for vesicle formation (Grant and Donaldson, 2009). Subsequently, Arf6 inactivation promotes sorting to early endosomes or recycling pathways. Other CIE pathways, including FEME, UFE and CLIC/GEEC also utilize small GTPases, but are described separately due to the larger number of additional proteins involved.

#### 1.1.4 CLIC/GEEC

Several CIE mechanisms operate independent of clathrin and dynamin, including CLIC/GEEC, which internalizes bulk fluid-phase material and glycosylphosphatidylinositol-anchored proteins (GPI-APs) in tubular carriers (Sabharanjak et al., 2002; Kirkham et al., 2005). At cholesterol-enriched PM microdomains, recruitment of the Arf1 guanine nucleotide exchange factor (GEF) GBF1 leads to Arf1 activation (Römer et al., 2010). Arf1-GTP subsequently recruits the Cdc42 GTPase-activating protein (GAP) ARGHGAP10/21, which regulates formation of endocytic carriers (Gupta et al., 2009). The Cdc42 effector GRAF1, which contains RhoGAP, SH3 and BAR-domains, also aids in membrane deformation while regulating Cdc42 activity (Lundmark et al., 2008). Additionally, CLIC/GEEC internalizes Shiga and cholera toxins, and may be activated to compensate for loss of membrane tension (Ferreira and Boucrot, 2018; Thottacherry et al., 2019).

#### 1.1.5 Fast endophilin-mediated and ultrafast endocytosis

FEME is a CIE pathway that relies on the BAR protein Endophilin to internalize cargos including G protein-coupled receptors (GPCRs), receptor tyrosine kinases (RTKs) and cytokine receptors (Boucrot et al., 2015). Upon ligand-receptor binding, Cdc42 initiates a signaling cascade via FBP17 and CIP4, which recruit the lipid phosphatases SHIP1/2; RhoA and Rac1 may also participate. Dephosphorylation of PI(3,4,5)P_3_ into PI(3,4)P_2_ permits anchoring of Lamellipodin, which binds to Endophilins A1 and A2 to facilitate membrane deformation (Hak et al., 2018). Finally, microtubules and microtubule-based motors are required for membrane tubule extension, while dynamin completes scission (Renard et al., 2015). Related to FEME is UFE, which involves endophilin-A3, synaptojanin-1, dynamin, and formin to mediate rapid recycling of synaptic membranes in neurons (Watanabe et al., 2013; 2018; Soykan et al., 2017; Imoto et al., 2022). FEME and UFE share some, but not all components, suggesting distinctions between the two pathways (Figure 1).

### 1.2 CIE in model organisms

Genetically-tractable model organisms provide important opportunities to further our understanding of endocytosis and its regulation. Notably, budding and fission yeast (*Saccharomyces cerevisiae* and *Schizosaccharomyces pombe*, respectively) were extensively used to uncover conserved CME proteins and to identify the sequence of events required for CCV formation (Engqvist-Goldstein and Drubin, 2003; Kaksonen et al., 2005; Yarar et al., 2005; Basu and Chang, 2011). Metazoan models such as *Drosophila melanogaster* and *Caenorhabditis elegans* are valuable for characterizing roles for endocytosis during development and in the context of tissue and organ function.

While model organisms aid in understanding CME, their use in studying CIE pathways remains limited by comparison. For example, CIE in budding yeast was first described in 2011; all yeast were previously thought to utilize only CME, even though residual endocytosis still occurs in clathrin-null cells (Payne et al., 1988; Prosser et al., 2011). In the following section, we describe recent breakthroughs in CIE from the standpoint of model organisms and how they provide insight into mammalian pathways.

#### 1.2.1 CIE in fungi

Similar to the mammalian RhoA-dependent pathway, CIE in budding yeast requires its homolog Rho1, as first described in CME-defective cells lacking functional adaptor proteins (Prosser et al., 2011)*.* In numerous CME-deficient mutants, including clathrin-null cells, high-copy expression of *RHO1*, its GEF *ROM1*, and the integrin-like cell wall stress sensor *MID2* improve cargo internalization, but do not correct aberrant dynamics of CME sites. Yeast CIE additionally requires the formin Bni1 (a Rho1 effector), polarisome proteins involved in Bni1 localization, and actin-stabilizing tropomyosins. Subsequent studies demonstrated CIE roles for select proteins involved in CME, including α-arrestins and Syp1, although their function in CME *versus* CIE may be mechanistically distinct (Prosser et al., 2015; Apel et al., 2017). Recent work demonstrated roles for the myosin Myo2, Myo2-dependent transport of cytoplasmic microtubules, microtubule-based motors (dynein/dynactin), and proteins involved in cortical microtubule capture (Num1) (Woodard et al., 2023). These findings suggest parallels between yeast CIE and mammalian pathways, including UFE (formin-dependent), FEME and uptake of cholera and Shiga toxins (microtubule and dynein/dynactin-dependent) (Day et al., 2015; Renard et al., 2015; Soykan et al., 2017).

Growing evidence suggests that CIE occurs in other fungi, although pathways remain poorly characterized. Fission yeast utilizes CME for endocytosis at cell tips, while formin (For3)-dependent actin polymerization facilitates internalization at the sides of cells (Gachet and Hyams, 2005); roles for clathrin in For3-dependent endocytosis have not yet been assessed. In *Candida albicans,* endocytosis persists in cells lacking Arp2/3, suggesting a likely CIE mechanism (Epp et al., 2013). *RHO1* overexpression does not restore endocytosis in *arp2/3* mutant *Candida*, suggesting differences from budding yeast CIE. Finally, in the filamentous fungus *Aspergillus nidulans*, an AP-2 and clathrin-independent endocytic pathway contributes to apical growth. Unlike budding yeast, α-arrestins are not involved in *Aspergillus* CIE, further suggesting mechanistic differences amongst fungi (Martzoukou et al., 2017).

#### 1.2.2 CIE in invertebrates

*Drosophila* is widely used for understanding the genetic basis of metazoan development, which requires coordination of signaling events dependent on receptor internalization. One example is Delta-Notch signaling, wherein Notch is endocytosed and induces cell proliferation and differentiation. Some Notch and Delta internalize in clathrin mutant flies in a dynamin-dependent manner, suggesting a CIE pathway of unknown mechanism (Windler and Bilder, 2010; Hemalatha et al., 2016). Moreover, *Drosophila* utilizes phagocytic, macropinocytic and CLIC/GEEC pathways for internalization (Gupta et al., 2009).

Several CIE pathways exist in *C. elegans,* including an Arf6-mediated pathway similar to the mammalian mechanism described above. This pathway contributes to sorting and recycling using Rab10, Rab22, Rab35, Hook1, ALX1 and RME-1/EHD-1 (Chen et al., 2006; Glodowski et al., 2007; Shi et al., 2007). In addition, *C. elegans* is useful for studying UFE, which was first described in worms. Upon light stimulation of motor neurons expressing channelrhodopsin, endocytic events begin 50 ms after stimulation and are completed within one second, faster than occurs during CME (Watanabe et al., 2013). Further study of this pathway showed that dynamin, endophilin, and synaptojanin are required, while clathrin is not.

#### 1.2.3 CIE in plants

Characterization of endocytosis in plants is limited, but studies in *Arabidopsis thaliana* demonstrate that CIE functions during environmental stress and seedling development. *Arabidopsis* CIE relies on Flotillin1 (Flot1) at sterol- and sphingolipid-enriched membrane microdomains distinct from clathrin-containing PM regions (Li et al., 2012). Additionally, Latrunculin B-mediated actin depolymerization in root tip cells inhibits formation of Flot1-positive compartments, suggesting actin involvement. Knockdown of Flot1 impairs seedling development and growth, implying roles for Flot1 and CIE in these processes (Li et al., 2012). *Arabidopsis* offers a unique tool for studying endocytosis in a whole tissue context due to high levels of organization in root cells and ease of visualization by light microscopy. Analysis of root tips demonstrates that GPI-APs and FM4-64 are constitutively internalized through CME and CIE in epidermal cells, while inner cells only require CME. However, under high salinity, CIE is upregulated across all root cells to internalize GPI-APs, transmembrane proteins, and FM4-64. This pathway is distinct from constitutive CIE, and requires the Rab5 GEF Vps9a, and sterols (Baral et al., 2015).

## 2 Discussion: Future directions in CIE

In recent years, our understanding of CIE has expanded to reveal diverse pathways; this increased knowledge opens new questions and avenues for future exploration. First, what is the degree of overlap between pathways? While some proteins clearly participate in both CME and CIE, or in multiple CIE pathways, contributions to different pathways may be mechanistically distinct. Often, different pathways share several major components, but recruit unique proteins to work with the core machinery. For example, the CLIC/GEEC pathway, FEME and UFE all involve Cdc42 during early events to activate proteins that modulate phosphoinositides, which bind BAR-domain proteins. BAR proteins differ between pathways, with CLIC/GEEC relying on GRAF1, FEME on endophilin-A1/A2 and UFE on endophilin-A3 (Lundmark et al., 2008; Sathe et al., 2018; Casamento and Boucrot, 2020). Among these, only CLIC/GEEC is dynamin-independent, suggesting distinct scission mechanisms. This mosaic pattern of components is common in CIE, and provides targets to dissect mechanistic commonalities and differences.

The lack of defined coat in CIE raises questions about how forces are generated for membrane bending. BAR-domain proteins sense and induce membrane curvature and may thus contribute to deformation, although how these proteins function in CIE remains unclear. Newly-described roles for myosins, microtubules and other cytoskeletal components in budding yeast CIE could similarly contribute to membrane deformation. In CME, the type I myosins Myo3/5 generate force during endocytosis through interactions with actin (Sun et al., 2006). Myo2 may play a similar role in CIE; alternatively, Myo2-dependent transport of microtubules to the cell cortex may suggest involvement of dynein/dynactin (Woodard et al., 2023).

Sorting and selection mechanisms are also unresolved questions, in large part because of the lack of CIE cargos. While cargos are known for RhoA-mediated endocytosis (IL2R) and CLIC/GEEC (GPI-APs), they remain less defined for other routes such as FEME and UFE. In yeast, the pheromone receptor Ste3 was used to characterize CIE, but prefers CME as its primary route (Prosser et al., 2011). Ptr2 is another yeast cargo that prefers CME at low osmolarity, but is partially redirected into a CIE path under high osmolarity conditions (Apel et al., 2017). Aside from these, new studies continue to reveal additional cargos that will likely allow us to better understand selection mechanisms during CME and CIE. For example, amyloid precursor protein (APP) and low-density lipoprotein internalize when clathrin and dynamin are inhibited, suggesting roles for an as-yet undetermined CIE route (Aow et al., 2022). Similarly, uptake of L1CAM and CD166 through Endophilin-A3-mediated CIE may facilitate studies of pathways such as FEME or UFE (Renard et al., 2019; Lemaigre et al., 2022).

As our knowledge of CIE expands, we are only beginning to understand these unique and exciting pathways. Much work remains in characterizing the proteins involved, elucidating physiological roles for CIE in health and disease, and assessing relationships between distinct CIE routes or between CME and CIE. Addressing these knowledge gaps is critical to expanding our understanding of CIE, and will provide important insights into the multiple mechanisms that contribute to PM regulation.
